# Biobased Composites of Poly(Lactic Acid) Melt Compounded with Bacterial and Vegetal Nanocelluloses Incorporated through Different Strategies

**DOI:** 10.3390/polym16070898

**Published:** 2024-03-25

**Authors:** Jimena Bovi, Juan Francisco Delgado, Orlando de la Osa, Mercedes Ana Peltzer, Celina Raquel Bernal, María Laura Foresti

**Affiliations:** 1 Universidad de Buenos Aires, Facultad de Ingeniería, Buenos Aires C1127AAR, Argentina; jbovi@fi.uba.ar (J.B.); jfdelgado@fi.uba.ar (J.F.D.); cbernal@fi.uba.ar (C.R.B.); 2 CONICET—Universidad de Buenos Aires, Instituto de Tecnología en Polímeros y Nanotecnología (ITPN), Buenos Aires C1127AAR, Argentina; 3 Universidad Nacional de Quilmes, Departamento de Ciencia y Tecnología, Laboratorio de Obtención, Modificación, Caracterización y Evaluación de Materiales (LOMCEM), Quilmes B1876BXD, Argentina; odelaosa@unq.edu.ar (O.d.l.O.); mercedes.peltzer@unq.edu.ar (M.A.P.); 4 Consejo Nacional de Investigaciones Científicas y Técnicas (CONICET), Buenos Aires C1425FQB, Argentina

**Keywords:** poly(lactic acid), composites, bacterial nanocellulose, rice husk nanocellulose, filler incorporation strategies

## Abstract

In the current contribution, bacterial nanocellulose obtained from a by-product of Kombucha tea production and vegetal nanocellulose isolated from milled rice husks were employed as fillers of PLA-based composites prepared by intensive mixing followed by compression molding. Given the challenges associated with the incorporation of nanocelluloses—initially obtained as aqueous suspensions—into melt compounding processes, and also with achieving a proper dispersion of the hydrophilic nanofillers within PLA, three different nanofibrils incorporation strategies were studied: i.e., direct mixing of dried milled nanocelluloses and PLA; masterbatching by solvent casting of native nanocelluloses followed by melt compounding; and masterbatching by solvent casting of acetylated nanocelluloses followed by melt compounding. Composites with varying filler content (from 0.5 wt.% to 7 wt.%) were characterized in terms of morphology, optical properties, and mechanical performance. Results revealed the relative suitability of each strategy employed to promote nanocelluloses dispersion within the PLA matrix. PLA/nanocellulose masterbatches prepared by solvent casting proved to be particularly useful for feeding the nanocelluloses into the processing equipment in a dry state with limited hornification. Acetylation also contributed to a better dispersion of both nanocelluloses within the PLA matrix, although no clear positive impact on the mechanical properties of the films was observed. Finally, filler loading played an important role in the films’ properties by increasing their stiffness while reducing their translucency.

## 1. Introduction

In the recent decades, the persistence of conventional non-biodegradable plastics in the environment has emerged as a global concern; and biodegradable polymers have garnered considerable attention. Currently, one of the most popular biodegradable polymers commercially available is poly(lactic acid) (PLA), a compostable thermoplastic polyester derived from biomass whose price has significantly declined over the last two decades [1,2]. 

Despite the relatively high mechanical strength and stiffness of poly(lactic acid), its gloss, and UV stability, PLA exhibits some disadvantages such as brittleness, a low crystallization rate, and relatively low barrier properties. In this regard, a well-established strategy to improve the performance of PLA is the development of composites with different types and amounts of fillers. Among them, in recent years, different biofillers have been assayed, with those of cellulosic nature being the most studied ones [3,4,5]. In particular, a huge variety of cellulosic nanomaterials has been employed [6,7]. Nanocelluloses are recognized for their numerous attractive attributes such as biodegradability, their non-toxic nature, the wide availability and renewability of the cellulosic sources, their lightweight nature, and impressive stiffness and strength, among others [6]. 

Over the last two decades, great advances in the development of PLA/nanocellulose composites prepared by solution casting have been achieved [8,9]. However, even if casting/evaporation-based processing techniques facilitate good dispersion of the nanofillers in the matrix, the amount of solvent and the time required usually limit their industrial scaling, so conventional melt processing techniques are preferred for large-volume production [10]. Nevertheless, achieving a proper nanofiller dispersion through melt compounding of nanocelluloses (especially cellulose nanofibrils) and PLA is much more challenging. Furthermore, cellulose nanofibrils are obtained and commonly commercialized as diluted aqueous suspensions, so their dehydration is highly desirable to avoid PLA degradation during thermal melting. However, upon water removal, extensive hydrogen bonding between cellulose nanofibrils is promoted, leading to their irreversible aggregation in a process known as hornification [11]. In the absence of superficial cellulose–water interaction, additional hydrogen bonds are created between adjacent cellulose nanofibrils, preventing further dispersion in aqueous or organic media [10]. In particular, oven-drying of cellulose nanofibrils is a great challenge since the hornification extent caused by this technique is usually greater than that induced by other drying methods [12,13]. Accordingly, direct oven-drying of cellulose nanofibrils prior to melt compounding with PLA usually hinders the later dispersion of nanocellulosic fillers within the polymeric matrix [14,15]. Moreover, the poor interfacial compatibility between the hydrophilic surface of nanocelluloses and the hydrophobic PLA further hampers the dispersion of cellulose nanofibrils. 

In this context, in recent years, a few contributions have proposed the elaboration of masterbatches by solvent casting as a middle step in the development of PLA/nanocelluloses melt-compounded composites with suitable levels of nanofiller dispersion [16,17]. In those masterbatches, PLA acts as a carrier to feed the nanocellulose into the processing equipment in a dry form, with PLA chains placed between cellulose nanofibrils, limiting hornification. On the other hand, aiming to improve the chemical compatibility between PLA and nanocelluloses, different strategies for the surface hydrophobization of the nanofibrils have been proposed, including polymer grafting, silanization, esterification (mainly acetylation), etc. [18]. 

In the current contribution, bacterial nanocellulose obtained from a by-product of Kombucha tea production (BNC) and vegetal nanocellulose isolated from milled rice husks (VNC) were employed as fillers of PLA-based composites prepared by intensive mixing followed by compression molding. Given the challenges associated with achieving a proper dispersion of cellulose nanofibrils—initially obtained as aqueous suspensions—within the PLA matrix by melt processing, three different filler incorporation strategies were assayed. In the first place, oven-dried milled bacterial and vegetal nanocelluloses were directly mixed with PLA. Secondly, masterbatches with native BNC and VNC were prepared by solvent casting, diluted with PLA pellets, and melt compounded. Finally, the masterbatches were elaborated by the same protocol, but using acetylated bacterial and vegetal nanocelluloses (AcBNC and AcVNC, respectively). Composites with varying filler contents were characterized and compared in terms of morphology, optical properties, and mechanical performance. 

## 2. Materials and Methods

### 2.1. Materials

Commercial PLA (Nature Works^®^ trade name 4043D) consisting of 6% D-lactide and 94% L-lactide, with a density of 1.24 g/cm^3^ and a melt flow index of 6 g/10 min (210 °C, 2.16 kg), was employed as the matrix of the composites. Before melt compounding, PLA pellets were vacuum dried at 80 °C for 4 h, as recommended by the supplier. Black tea (Green Hills, Buenos Aires, Argentina) and glucose (Xantana, Buenos Aires, Argentina) were used in the formulation of culture media for the production of bacterial nanocellulose. The SCOBY (Symbiotic Culture of Bacterial and Yeast) was kindly supplied by Aloha Fermentos (Buenos Aires, Argentina). Rice husks remaining from rice production (Chajarí, Entre Ríos, Argentina) were washed, micronized, and sieved to retain particles smaller than 125 µm. Hydrochloric acid (Biopack, Buenos Aires, Argentina), sodium hydroxide (Anedra, Buenos Aires, Argentina), and sodium chlorite solution (Anedra, Buenos Aires, Argentina) were employed for the isolation of vegetal cellulose, whereas potassium hydroxide (Cicarelli, Santa Fe, Argentina) was used in the purification of bacterial nanocellulose. Acetic acid, acetic anhydride, acetone, and chloroform (all from Cicarelli, Santa Fe, Argentina) were used for nanocellulose acetylation and for the preparation of the masterbatches. All chemicals used were of analytical grade and employed as received. 

### 2.2. Bacterial Nanocellulose Production

Bacterial nanocellulose was isolated from the by-product of Kombucha tea production, as described previously [19]. Briefly, a black tea infusion (10 g/L in boiling water) was sweetened with 60 g/L of glucose. After cooling, the inoculum was added at a concentration of 10% *v*/*v*, and covered containers were kept in static conditions at 28–30 °C for 14 days. After this period, the floating nanocellulose membranes developed on the surface of the broth were harvested, thoroughly rinsed with distilled water to remove the culture medium, broken down for 4 min using a kitchen blender in a KOH 5% (*w*/*v*) solution, left in alkali for 16 h at room temperature, and successively rinsed with distilled water. 

### 2.3. Vegetal Nanocellulose Production

Sieved milled rice husks (particle size < 125 µm) were put in contact (0.05 g/mL) with a sodium hydroxide solution (4 wt.%), refluxed for 2 h with magnetic stirring, exhaustively washed with distilled water, and filtered. The solid obtained was then treated (0.03 g/mL, dry weight) with a sodium chlorite solution (1.7% *w*/*v*, in acetic acid/sodium acetate/distilled water buffer, pH 4) at 90 °C for 2 h under reflux, followed by extensive washing with distilled water. The resulting cellulosic fraction was treated (0.04 g/mL) with a hydrochloric acid solution (1 M) at 90 °C for 45 min, and then washed again with distilled water until the washing waters reached the pH of distilled water [20]. The cellulose suspension (1 wt.%) was subsequently passed through a high-pressure homogenizer NS1001L (Niro-Soavi, Parma, Italy) operated at 100 MPa for 1 h.

### 2.4. Acetylation of Nanocelluloses

Aqueous suspensions BNC and VNC (1 wt.%) were solvent exchanged through acetic acid into acetic anhydride (twice each, using 200 mL). Vacuum-filtrated BNC and VNC (2.5 g dry weight) were then transferred into a glass flask containing 250 mL of acetic anhydride. The system, equipped with a reflux condenser, was heated at 120 °C for 1 h in a thermostatized oil bath under continuous magnetic stirring. After this period of time, the obtained AcBNC and AcVNC were vacuum-filtrated and thoroughly washed with ethanol and distilled water. The attained degrees of substitution (DS) of both modified nanocelluloses were quantified by heterogeneous saponification of dried samples according to Ávila Ramírez et al. (2014) [21].

### 2.5. Masterbatches and Composites Preparation

In the first strategy assayed, homogenized nanocelluloses (18,000 rpm, 5 min, 5 times) were dried (110 °C, 24 h), milled, and directly introduced into a Brabender internal mixer (170 °C, 50 rpm, 8 min) with the required amount of PLA pellets to reach filler concentrations of 0.5, 1, 1.5, 3, 5, and 7 wt.%. The composites obtained with this strategy were named PLA/BNC-D and PLA/VNC-D. For comparison, neat PLA was also melt compounded using the same processing conditions. As a second strategy, masterbatches (MBs) were prepared by solvent casting. For this purpose, aqueous suspensions of native bacterial and vegetal nanocelluloses were solvent-exchanged through acetone (twice) into chloroform (twice) with homogenization (18,000 rpm, 5 min). Subsequently, nanocellulose suspensions were homogenized in chloroform (65 mL) containing dissolved PLA at proper concentrations to achieve masterbatch filler contents of 16.7 wt.%, cast onto glass vessels covered with Teflon tape, and dried (room temperature, 48 h; followed by 60 °C in a vacuum oven, 2 weeks). Then, the masterbatches were milled, dried under the same conditions as additional PLA pellets (80 °C, under vacuum, 4 h), and melt compounded in a Brabender internal mixer (170 °C, 50 rpm, 8 min) to reach final filler concentrations of 0.5, 1, 1.5, 3, 5, and 7 wt.%. The composites obtained using this strategy were named PLA/BNC-MB and PLA/VNC-MB. The third strategy was the same as the second one, but acetylated bacterial nanocellulose and acetylated vegetal nanocellulose instead of their native counterparts were used as fillers. The resulting composites from this last strategy were named PLA/AcBNC-MB and PLA/AcVNC-MB.

To obtain the films, 5.5 g of the melt-compounded samples (PLA and each of the described composites) previously dried at 80 °C under vacuum overnight were compression molded using a hydraulic press model 3853 (Carver, Wabash, IN, USA) at 170 °C without any pressure, 8 min, followed by 170 °C at 2 MPa for 5 min, and cooling at room temperature. The thickness of all composites ranged from 0.6 to 0.9 mm, and their formulations are summarized in Table 1.

### 2.6. Characterization of Nanocelluloses 

#### 2.6.1. Field Emission Scanning Electron Microscopy (FESEM)

Drops of aqueous suspensions (0.01 wt.%) of BNC, VNC, AcBNC, and AcVNC were placed on microscope slides, dried at 110 °C for 5 min, sputter coated with gold, and observed using a Zeiss Crossbeam 340 FESEM microscope (Carl Zeiss Microscopy GmbH, Oberkochen, Germany) operated at an accelerating voltage of 3 kV. Quantitative image analysis was performed with the help of the image processing software Image J 1.53e. In order to guarantee the statistical validity of the analysis, at least 200 measurements were performed.

#### 2.6.2. Atomic Force Microscopy (AFM)

Drops of aqueous suspensions (0.2 wt.%) of BNC, VNC, AcBNC, and AcVNC were placed on a mica support and analyzed using a Nanoscope V-Multimode atomic force microscope (Veeco, Santa Barbara, CA, USA) in tapping mode (intermittent contact) using a silicon nitride probe Arrow-NCR-50 (Nano World, Neuchâtel, Switzerland). The resonance frequency of the cantilever was 258 kHz, with a constant force of 42 N m^−1^ and a tip radius ranging from 5 to 10 nm. Scanning frequencies typically ranged from 1.0 to 1.5 Hz. 

#### 2.6.3. Fourier Transform Infrared Spectroscopy (FTIR)

Fourier transform infrared spectra of BNC, VNC, AcBNC, and AcVNC were acquired with an IR Affinity-1 Shimadzu Fourier Transform Infrared Spectrophotometer (Kyoto, Japan) in absorbance mode. Dried milled samples and KBr (110 °C, overnight) were pressed at a 1:20 ratio into discs at 8 kg/cm^2^ and the IR spectra were recorded in the range of 3800 to 900 cm^−1^ with a total of 32 scans at a resolution of 4 cm^−1^. Baselines were corrected and normalized against the intensity of the absorption at 1165 cm^−1^, attributed to the (C–O–C) linkage of cellulose [22]. 

#### 2.6.4. X-ray Diffraction Analysis (XRD)

A Rigaku D/Max-C wide-angle automated X-ray diffractometer with vertical goniometer was employed to collect the X-ray diffraction patterns of BNC, VNC, AcBNC, and AcVNC (Cu/Kα, 0.154 nm, 40 kV, 30 mA). Diffractograms were recorded at a step size of 0.02° in the 2θ range of 10–50°. For the calculation of the crystallinity index of all samples, Segal’s empirical Equation (1) was applied [23]:

(1) $$CrI\;\left(\%\right)=\frac{I_{002}-I_{am}}{I_{002}}\;*100\%\;$$

where I_002_ is the maximum intensity value of the 002 lattice diffraction and represents crystalline and amorphous material, and I_am_ is the intensity at 2θ = 18° and represents amorphous material only. 

### 2.7. Characterization of PLA/Nanocelluloses Composites

#### 2.7.1. Environmental Scanning Electron Microscopy (ESEM)

Cryo-fractured surfaces of PLA and PLA/nanocelluloses composites obtained at liquid nitrogen temperature were analyzed using an FEI Quanta 200 microscope operated at an accelerating voltage of 15 kV. All samples were sputter-coated with a thin layer of gold before observations. 

#### 2.7.2. Optical Properties

Light transmittance curves of PLA and PLA/nanocelluloses composites were obtained in the range of 200–800 nm using a Shimadzu UV-visible spectrophotometer (Model UV-1650pc). The color of the films was evaluated using a Minolta colorimeter (CR-20, Konica Minolta, Inc., Tokyo, Japan) previously calibrated with a white reflector plate. Measurements were expressed as colorimetric coordinates in the CIELAB scale: L* (lightness, from 0: black to 100: white), a* (from green (−) to red (+)), and b* (from blue (−) to yellow (+)). Color differences (ΔE) were calculated using Equation (2):

(2) $$\Delta E=\sqrt{{\Delta L*}^{2}+{\Delta a*}^{2}+{\Delta b*}^{2}}$$

#### 2.7.3. Mechanical Properties

Uniaxial tensile tests were performed on PLA and PLA/nanocellulose composites using dumbbell samples (at least five per system) in an INSTRON dynamometer 5985 (Norwood, MA, USA) at 5 mm/min, using a load cell of 1 kN [24]. Young’s modulus, tensile strength, and strain-at-break values were obtained from the stress–strain curves. Average values of tensile parameters were reported along with their deviations.

## 3. Results and Discussion

### 3.1. Nanocelluloses

**Figure 1:**
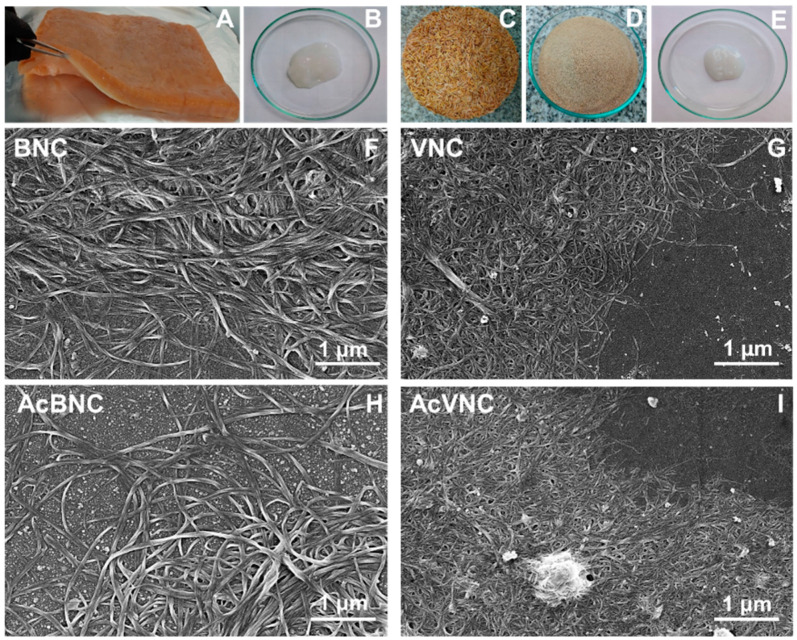
Photographs of the unpurified floating pellicle developed during Kombucha tea production (**A**), bacterial nanocellulose recovered after purification of the pellicle (**B**), rice husks washed (**C**) and milled (**D**), and vegetal nanocellulose isolated from rice husks (**E**). FESEM micrographs of diluted aqueous suspensions of BNC (**F**), VNC (**G**), AcBNC (**H**), and AcVNC (**I**).

Figure 1 shows collected photographs of the raw materials used for nanocellulose isolation, as well as the purified bacterial and vegetal celluloses (i.e., BNC and VNC, respectively). FESEM micrographs of BNC, VNC, AcBNC, and AcVNC are also shown. Figure 1A is a photograph of the floating pellicle developed during Kombucha production, while Figure 1B shows the purified BNC isolated according to Section 2.2. Melanoidins present in the Kombucha tea give the pellicle a brownish color. Blending in an alkali solution promotes the removal of these components, resulting in the gel-like material with a whitish color shown in Figure 1B. Figure 1C,D present the original and the milled rice husks, respectively. According to the literature, rice husks are composed of cellulose (25–35 wt.%), hemicellulose (18–21 wt.%), lignin (26–31 wt.%), silica (15–17 wt.%), and other soluble compounds (2–5 wt.%) [25,26]. Figure 1E shows the appearance of the VNC obtained after purification and fibrillation of cellulose from rice husks as described in Section 2.3.

FESEM images collected in Figure 1F–I illustrate the morphology of the native and acetylated nanocelluloses obtained. In BNC and AcBNC images, the typical intertwisted bacterial cellulose nanoribbons with an average width of 40 ± 10 nm and several micrometers in length can be distinguished, in agreement with values reported in the literature [27,28]. Nanocelluloses derived from rice husks showed an average width of 27 ± 6 nm, also in accordance with previous reports [17,29]. Regarding VNC and AcVNC length, even if micrometric nanofibrils seem to prevail, some shorter fibrils can be distinguished in less concentrated zones. 

**Figure 2:**
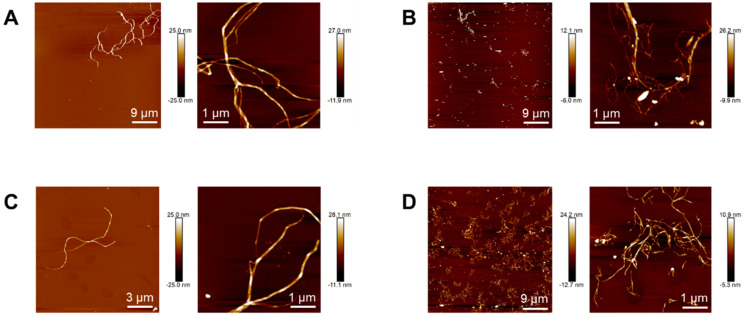
Topographical images of BNC (**A**), VNC (**B**), AcBNC (**C**), and AcVNC (**D**) acquired by atomic force microscopy.

Topographical images of nanocelluloses obtained by atomic force microscopy allowed better estimating their length. Whereas native and acetylated bacterial nanocelluloses (Figure 2A and Figure 2C, respectively) show nanofibrils with lengths between 3–15 μm, in native and acetylated vegetal nanocelluloses images (Figure 2B and Figure 2D, respectively) micrometric-in-length nanofibrils seem to coexist with a significant fraction of much shorter fibrils (up to 200 nm long). The latter observations suggest that the final step during VNC isolation involving hydrochloric acid might have promoted the reduction in the length of a fraction of the fibrils. Similar results were reported in the isolation of cellulose nanofibrils from other vegetal sources using protocols that involved treatments with hydrochloric acid solutions under comparable conditions (usually aimed at removing compounds other than cellulose [30] and/or improving fiber exfoliation [31]).

**Figure 3:**
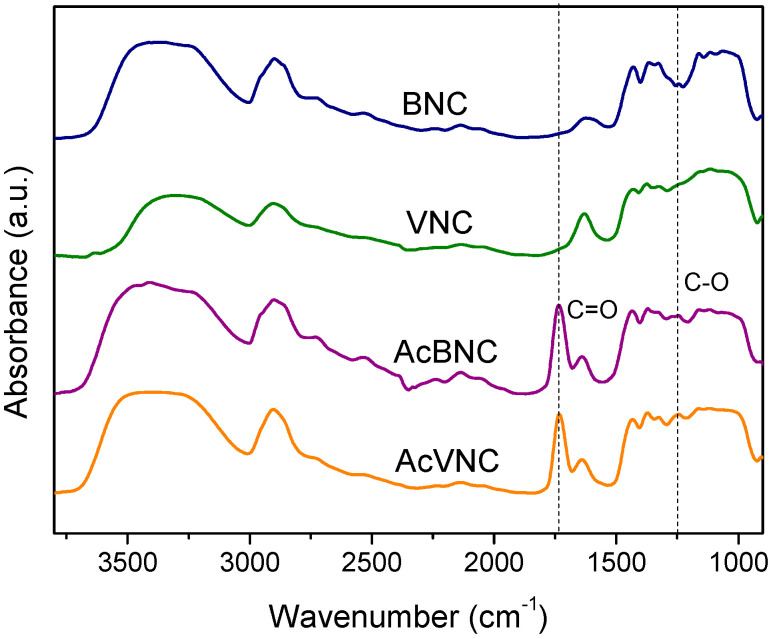
FTIR spectra of BNC, VNC, AcBNC, and AcVNC. Curves were shifted along the y-axis for clarity.

The FTIR spectra of native and acetylated bacterial and vegetal nanocelluloses are shown in Figure 3. All samples exhibit absorbances typical of cellulose, i.e., those found in the 3600–3000 cm^−1^ interval ascribed to the stretching vibration of -OH groups present in carbohydrates; bands found in the 3000–2800 cm^−1^ region attributed to the stretching of C-H linkages of methyl and methylene groups of cellulose; and other signals within the 1450–900 cm^−1^ range, commonly associated with the symmetrical bending of CH_2_ group, cellulose C-O-C bridges, C-O stretching and other absorptions typical of β-linked glucose polymers [27,32,33]. In addition, both acetylated nanocelluloses FTIR spectra present signals characteristic of ester groups (e.g., stretching of C=O at 1735 cm^−1^ and stretching of C-O at 1248 cm^−1^), confirming the occurrence of the modification [34,35]. For both acetylated nanocelluloses, the attained degree of substitution (DS) determined by saponification was in the range of 0.15–0.20.

**Figure 4:**
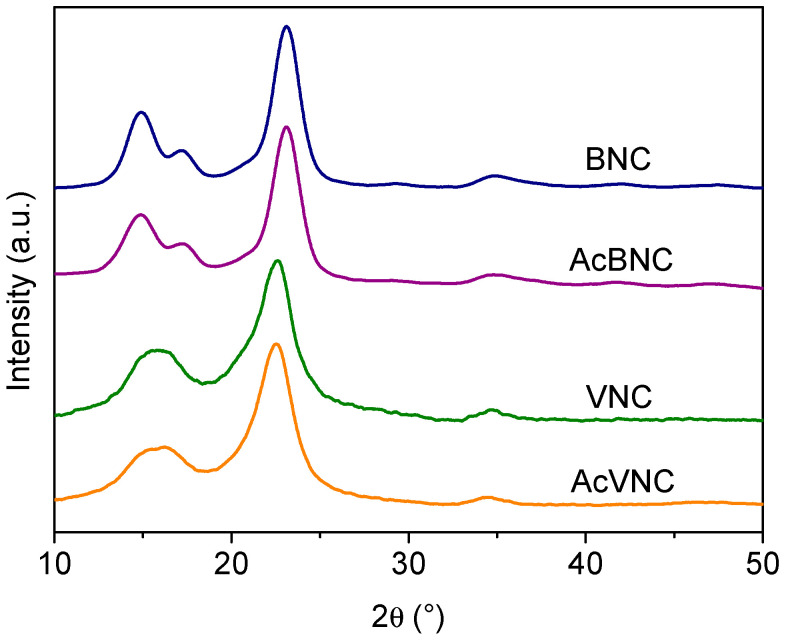
X-ray diffractograms of BNC, VNC, AcBNC, and AcVNC. Curves were shifted along the y-axis for clarity.

Figure 4 presents the X-ray diffractograms of the different samples. Four signals characteristic of the crystalline peaks of cellulose I can be observed in BNC and AcBNC samples centered at 2θ = 14.9°, 17.2°, 23.1°, and 34.9° in accordance with previous reports [35,36,37]. The fifth signal characteristic of cellulose I samples sometimes reported at 20.5° (usually a low-intensity signal) is not clearly defined in the bacterial nanocelluloses diffractograms. The crystallinity index (CrI) of BNC and AcBNC calculated by Segal’s empirical method was (85 ± 1) %, in agreement with previous values calculated for microbial nanocelluloses derived from Kombucha tea production using the same method [38,39]. 

In reference to VNC and AcVNC samples, three characteristic cellulose I intense signals are observed centered at 2θ = 15.8° (resulting from the overlapping of peaks centered at 2θ = 14.9° and 16.3°), 22.7°, and 34.6° [36]. The calculated crystallinity index for VNC and AcVNC was (76 ± 2) %, in accordance with previous reports for vegetal nanocelluloses [31,40], which are typically less crystalline than nanocelluloses of bacterial origin [41,42]. As described by other authors, the higher crystallinity of bacterial nanocelluloses is also illustrated in their better-resolved diffractograms [40,41,42]. 

On the other hand, the fact that the CrIs of both acetylated nanocelluloses were similar to those of their native counterparts suggests that chemical modification was limited to the surface and more accessible amorphous domains of the cellulose, and that the crystalline arrangement of the inner layers of the fibrils was not significantly altered upon acetylation [35,40,41].

### 3.2. PLA/Nanocelluloses Composites

**Figure 5:**
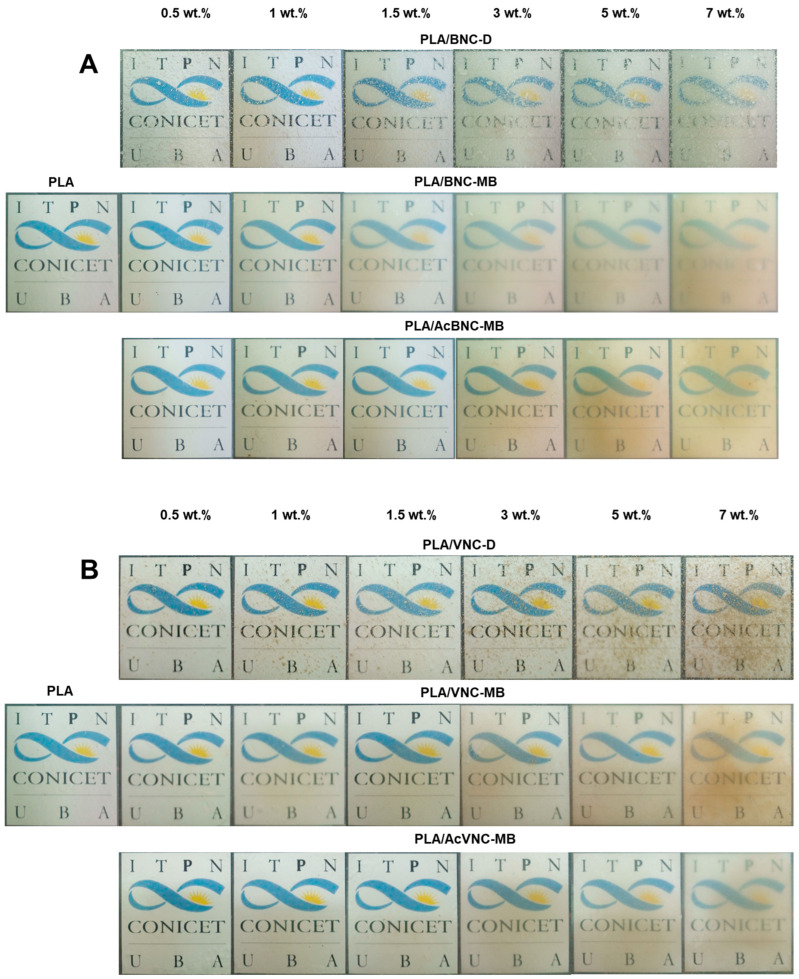
Photographs of neat PLA, PLA/BNC-D, PLA/BNC-MB, and PLA/AcBNC-MB (**A**), and PLA/VNC-D, PLA/VNC-MB, and PLA/AcVNC-MB (**B**) composite films obtained using different filler incorporation strategies and nanocelluloses contents.

Figure 5 collects representative photographs of neat PLA and all the composite films obtained by intensive mixing and compression molding with the fillers incorporated through the three different strategies assayed. The direct introduction of dried milled bacterial or vegetal nanocelluloses into the PLA matrix resulted in a poor dispersion of the filler (PLA/BNC-D and PLA/VNC-D in Figure 5A and Figure 5B, respectively), with the appearance of nanocellulose aggregates as dots of varying size all over the film. 

Aggregates of nanocelluloses smaller than 0.5 mm were mainly observed, although larger ones can be also distinguished (with sizes up to 4 mm in the case of PLA/BNC-D, and up to 2 mm for PLA/VNC-D), affecting the optical properties of the films. 

On the other hand, all composites obtained through masterbatching exhibit much smaller aggregates (especially in the case of BNC-based composites), highlighting an improved dispersion of the nanofillers. Results suggest that the strategy of preparing PLA/nanocellulose masterbatches by solvent casting successfully allowed feeding the nanocellulose into the processing equipment in a dry state with limited hornification by placing PLA chains between cellulose nanofibrils. Regarding the effects of acetylation, the images in Figure 5 show that chemical modification further improved the dispersion of both nanocelluloses within the PLA matrix compared to their native counterparts, according to a higher compatibility between the hydrophobic matrix and the hydrophobized nanocelluloses, as previously reported by other authors for similar systems [34,35,40]. Tingaut et al. (2010) evaluated the quality of filler dispersion within PLA using transparency as an indicator of good dispersibility. The authors highlighted the beneficial effect of microfibrillated cellulose acetylation on the reduction in the presence of aggregates [43]. The effect of filler loading is also evident from the images in Figure 5, with films showing reduced translucency as nanocellulose content was increased. Filler aggregation during films’ manufacturing compromises their optical properties, negatively affecting the product appearance [44].

**Figure 6:**
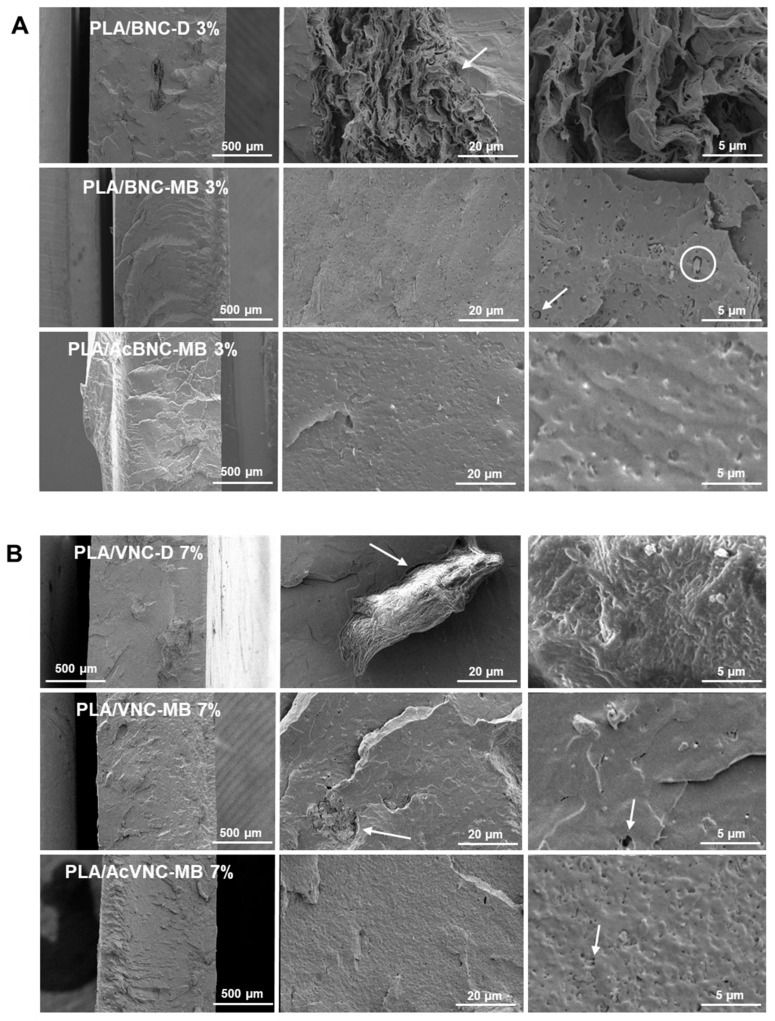
ESEM micrographs of cryo-fractured surfaces of PLA/BNC-D, PLA/BNC-MB, and PLA/AcBNC-MB composite films with 3 wt.% of bacterial nanocellulose (**A**), and PLA/VNC-D, PLA/VNC-MB, and PLA/AcVNC-MB composite films with 7 wt.% of vegetal nanocellulose (**B**). Circles and arrows were added to point gaps at the filler/matrix interface.

Representative cryo-fractured surfaces of PLA and PLA/nanocellulose composites are presented in Figure 6. Large irregular aggregates of nanocelluloses can be observed in the ESEM micrographs of composites obtained by the direct introduction of dried milled BNC and VNC into PLA (Figure 6A,B, upper rows). In accordance with the observations from Figure 5, BNC aggregates are larger than VNC ones and they have the typical multilayered arrangement of pure dried BNC films [45], with no evidence of PLA intercalation. Results are consistent with previous observations for composites obtained by mixing dried milled BNC and PLA [46,47]. The limited dispersion of the milled reinforcements can be attributed to the hornification of nanocelluloses taking place upon drying, as well as to the inherent incompatibility between components, resulting in a poor adhesion between phases as evidenced by the presence of gaps at the filler/matrix interface, some of them pointed to with arrows in Figure 6A,B, upper rows. Evident voids around cellulosic aggregates have previously been associated with poor adhesion between PLA and the filler [48,49].

Regarding composites obtained through masterbatching, a significant reduction in the size of the aggregates is observed (Figure 6A,B, middle rows), and in the case of BNC, the typical morphology of pure multilayered BNC aggregates is no longer distinguishable. This further indicates that the elaboration of a masterbatch by solvent casting before melt compounding succeeded in decreasing the hornification of both nanocelluloses and promoted a better dispersion of the fillers within the PLA matrix. Improved dispersion of nanocelluloses upon masterbatching is consistent with the results reported by Bagheriasl and co-workers, who evaluated the relative dispersion of cellulose nanocrystals introduced through direct mixing and masterbatching strategies into PLA matrix [50]. Regarding the effect of acetylation, no evident improvement was observed, with similar surface roughness and dispersion levels (Figure 6A,B, bottom rows) [34]. No significant effect of filler content was evidenced from ESEM fractographs either. 

**Figure 7:**
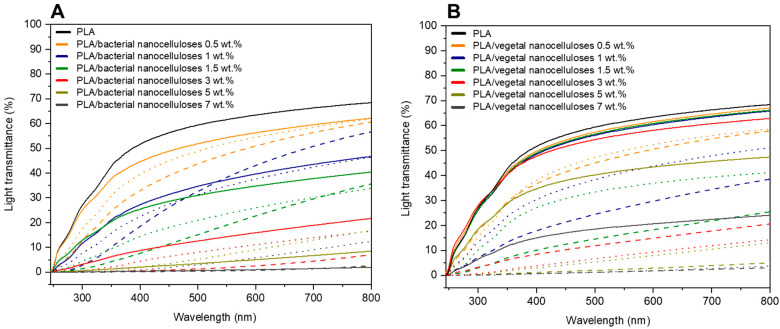
Light transmittance curves of neat PLA, PLA/BNC-D (solid lines), PLA/BNC-MB (dashed lines), and PLA/AcBNC-MB (dotted lines) (**A**); and PLA/VNC-D (solid lines), PLA/VNC-MB (dashed lines), and PLA/AcVNC-MB (dotted lines) (**B**) composite films obtained using different filler incorporation strategies and nanocellulose contents.

Figure 7 exhibits the light transmittance (%) curves of composite films collected in the range of 200–800 nm. For both nanocelluloses, and regardless of the strategy employed for their incorporation into the PLA matrix, Figure 7A,B show that composites displayed lower light transmittance values than PLA throughout the measured wavelength range. Additionally, in agreement with visible changes illustrated in Figure 5, light transmittance values significantly decreased as higher filler contents were used. The light transmittance of composites containing 5 wt.% and 7 wt.% of nanocellulose was especially low.

In terms of the effects of the strategies employed to incorporate the cellulosic nanofillers, most results collected in Figure 7 indicate that the direct introduction of both dried milled nanocelluloses resulted in composites with higher light transmittance values than composites produced by masterbatching, regardless of the filler content. Results may be attributed to the high hornification of dried milled nanocelluloses which, when directly introduced into the polymer melt, dispersed poorly, and composite materials with relatively extensive zones of the neat matrix with the high light transmittance characteristic of PLA were obtained. On the other hand, the preparation of composites by masterbatching through solvent casting promoted a better dispersion of less hornified nanocelluloses within the matrix, and the measured light transmittance values were the result of much more homogenous materials involving the PLA matrix and smaller nanocellulose aggregates. Regarding the effect of acetylation in composites prepared by masterbatching, in general, hydrophobized nanocelluloses led to composites with higher light transmittance curves than those involving native nanocelluloses, as previously reported for similar systems [34,35,40], and in accordance with observations from Figure 5. These results further suggest an improvement in the compatibility of nanocelluloses and PLA upon chemical modification superimposed to the reduced hornification of nanocelluloses derived from masterbatching.

Table 2 collects the color parameters of PLA and all composite films obtained. Compared to PLA, composite films show slightly reduced L*; similar a*; and higher b* values, resulting in ΔE values between 0.3 and 27.4. More pronounced changes in color parameters were observed as the filler content was increased, especially in more homogeneous composites prepared by masterbatching. Similar findings were reported by other authors who developed composites based on PLA reinforced with bacterial nanocellulose [51]. One of the most common applications of PLA-based materials is food packaging, where visual properties play a key role in influencing consumer preference. For this use, lighter and more transparent materials with less saturated colors are favored [52]. Accordingly, regarding color, the composites with lower filler contents developed herein (e.g., those with nanocellulose contents ≤ 1.5 wt.%) would be more suitable for those applications. 

**Figure 8:**
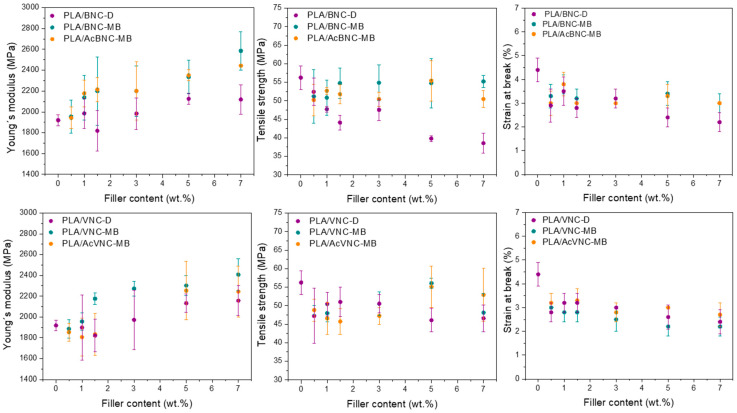
Mechanical properties of neat PLA, PLA/BNC-D, PLA/BNC-MB, PLA/AcBNC-MB, PLA/VNC-D, PLA/VNC-MB, and PLA/AcVNC-MB composite films obtained using different filler incorporation strategies and nanocellulose contents.

Tensile parameters values of neat PLA and the composites developed as a function of filler content and for the three strategies assayed are presented in Figure 8. Regardless of the type of nanocellulose used, most composites presented higher stiffness values than neat PLA, and an increasing trend of these values with nanocellulose content was observed as a result of the introduction of stiff fillers into a more compliant matrix [14]. In addition, an effect of the incorporation strategy was also evident in Young’s modulus values, with higher values for the composites obtained through masterbatching, and no significant effect derived from nanofiller acetylation. The previous preparation of masterbatches by solvent casting enhanced the dispersion of the nanofillers by hindering their hornification during drying from a diluted system in the presence of PLA chains. This effect was particularly pronounced in the case of the composites containing BNC and AcBNC. As Young´s modulus is a mechanical parameter obtained at low deformation levels, it is mostly determined by the dispersion of the filler within the matrix and should not be affected by filler–matrix interaction [53]. Enhanced dispersion of nanocelluloses within PLA derived from masterbatching justifies the stiffness increases observed.

On the other hand, the direct incorporation of dried milled nanocelluloses into PLA led to a reduction in the material’s strength. Tensile strength depends on the interaction between the components, which determines stress transfer from the matrix to the filler, and also on the filler dispersion within the matrix. The results obtained herein indicate an insufficient matrix ability for stress transfer to the hydrophilic fillers poorly dispersed in the hydrophobic polymer matrix [37,41]. A decreasing trend of strength with filler content was also observed—especially for bacterial nanocellulose composites—attributed to an increasing content of poorly dispersed filler. Alternatively, for VNC composites prepared by masterbatching, the neat PLA film strength value was restored at the highest filler loadings used (i.e., 5 wt.% and 7 wt.%), whereas the same strategy led to BNC composites with tensile strength values similar to that of PLA for all filler contents assayed. Compared to the composites produced by direct mixing of milled dried nanocelluloses with PLA, masterbatching led to stronger composites as a result of better nanofiller dispersion due to reduced hornification effects. Finally, with the aim to increase the interaction between both phases and further improve fillers dispersion, nanocelluloses were surface-acetylated prior to masterbatching. Although the dispersion of the fillers had been found to improve as a result of chemical modification (Figure 5 and Figure 7), no significant changes in tensile strength were observed, suggesting that the interfacial adhesion between PLA and acetylated nanocelluloses was not significantly improved at the degree of substitution conferred to the nanofillers (i.e., DS: 0.15–0.20).

Finally, the ductility of all composites was significantly reduced compared to neat PLA, and a slight decreasing trend was found as nanocellulose contents were increased. These findings are consistent with results reported by others for similar systems [34,48,54,55,56,57]. The reduction in ductility exhibited by the composites in comparison to neat PLA is commonly observed in thermoplastic composites [58] and can be attributed to the restriction imposed by a stiffer filler to matrix plastic deformation, with the decrease in ductility more noticeable at higher loadings.

## 4. Conclusions

In the current contribution, bacterial nanocellulose obtained from a by-product of Kombucha tea production and vegetal nanocellulose isolated from milled rice husks, both in native and acetylated forms, were employed as fillers in PLA-based composite films prepared by intensive mixing followed by compression molding. Given the challenges associated with the proper dispersion of nanocelluloses—initially obtained as aqueous suspensions—in PLA by melt compounding processing, three different nanofibrils incorporation strategies at six different nanofiller loadings were investigated. 

Morphological, optical, and mechanical characterization of the developed films revealed that the previous preparation of masterbatches by solvent casting successfully improved the dispersion of the nanocellulosic fillers within the polyester matrix compared to the direct mixing of dried milled nanocelluloses and PLA. These results were attributed to the limited hornification of the fillers achieved upon drying in the presence of PLA. The development of masterbatches by solvent casting from homogenized well-dispersed never-dried nanofibers suspensions involved drying nanocellulose suspensions in the presence of PLA chains which, according to the results obtained, must have interfered in the formation of intermolecular hydrogen bonding among cellulose fibrils, limiting hornification and promoting later easier dispersion of the nanofibrils in the final composites.

Regarding the effects of acetylation, chemical modification of nanocelluloses used in the masterbatches further improved the dispersion of both nanocelluloses within the PLA matrix, according to a higher compatibility between the hydrophobic matrix and the surface-hydrophobized nanocelluloses. Nonetheless, the enhanced dispersion of acetylated fillers did not lead to a clear improvement in the mechanical properties of the films. In particular, in reference to composite strength, the degree of substitution conferred to nanocelluloses may have not been enough to promote the interfacial adhesion required for effective stress transfer.

The influence of filler loading was also evident from the different characterization assays performed. Particularly important were its effects on increasing the materials’ stiffness while reducing their translucency.

Overall, the preparation of masterbatches by solvent casting as a previous step to melt processing appears to be a promising strategy for developing PLA/nanocelluloses composites, combining the high dispersibility advantages of lab-scale solvent casting with an industrially viable processing technique. It is worth noting that the strategies used were assayed in composites containing nanofillers isolated from wastes of other processes (Kombucha and rice production), but they may be extended to other nanocellulose sources.

## Figures and Tables

**Table 1:** PLA/nanocelluloses composite formulations.

Composite	PLA (wt.%)	BNC (wt.%)	VNC (wt.%)	AcBNC (wt.%)	AcVNC (wt.%)
PLA	100	-	-	-	-
PLA/BNC-D 0.5%	99.5	0.5	-	-	-
PLA/BNC-D 1%	99	1	-	-	-
PLA/BNC-D 1.5%	98.5	1.5	-	-	-
PLA/BNC-D 3%	97	3	-	-	-
PLA/BNC-D 5%	95	5	-	-	-
PLA/BNC-D 7%	93	7	-	-	-
PLA/VNC-D 0.5%	99.5	-	0.5	-	-
PLA/VNC-D 1%	99	-	1	-	-
PLA/VNC-D 1.5%	98.5	-	1.5	-	-
PLA/VNC-D 3%	97	-	3	-	-
PLA/VNC-D 5%	95	-	5	-	-
PLA/VNC-D 7%	93	-	7	-	-
PLA/BNC-MB 0.5%	99.5	0.5	-	-	-
PLA/BNC-MB 1%	99	1	-	-	-
PLA/BNC-MB 1.5%	98.5	1.5	-	-	-
PLA/BNC-MB 3%	97	3	-	-	-
PLA/BNC-MB 5%	95	5	-	-	-
PLA/BNC-MB 7%	93	7	-	-	-
PLA/VNC-MB 0.5%	99.5	-	0.5	-	-
PLA/VNC-MB 1%	99	-	1	-	-
PLA/VNC-MB 1.5%	98.5	-	1.5	-	-
PLA/VNC-MB 3%	97	-	3	-	-
PLA/VNC-MB 5%	95	-	5	-	-
PLA/VNC-MB 7%	93	-	7	-	-
PLA/AcBNC-MB 0.5%	99.5	-	-	0.5	-
PLA/AcBNC-MB 1%	99	-	-	1	-
PLA/AcBNC-MB 1.5%	98.5	-	-	1.5	-
PLA/AcBNC-MB 3%	97	-	-	3	-
PLA/AcBNC-MB 5%	95	-	-	5	-
PLA/AcBNC-MB 7%	93	-	-	7	-
PLA/AcVNC-MB 0.5%	99.5	-	-	-	0.5
PLA/AcVNC-MB 1%	99	-	-	-	1
PLA/AcVNC-MB 1.5%	98.5	-	-	-	1.5
PLA/AcVNC-MB 3%	97	-	-	-	3
PLA/AcVNC-MB 5%	95	-	-	-	5
PLA/AcVNC-MB 7%	93	-	-	-	7

**Table 2:** Color parameters of neat PLA, PLA/BNC-D, PLA/BNC-MB, PLA/AcBNC-MB, PLA/VNC-D, PLA/VNC-MB, and PLA/AcVNC-MB composite films obtained using different filler incorporation strategies and nanocellulose contents. ΔE values were calculated with respect to neat PLA.

Composite	L*	a*	b*	ΔE	Composite	L*	a*	b*	ΔE
PLA	80.8 ± 0.9	−0.2 ± 0.1	−1.5 ± 0.6	-	PLA	80.8 ± 0.9	−0.2 ± 0.1	−1.5 ± 0.6	-
PLA/BNC-D 0.5%	78.6 ± 1.5	−0.6 ± 0.5	−0.5 ± 0.4	2.5	PLA/VNC-D 0.5%	80.6 ± 0.5	−0.2 ± 0.0	−1.9 ± 0.3	0.0
PLA/BNC-D 1%	79.3 ± 0.6	−0.5 ± 0.0	−0.8 ± 0.3	1.7	PLA/VNC-D 1%	79.7 ± 0.5	−0.2 ± 0.0	−1.2 ± 0.3	1.1
PLA/BNC-D 1.5%	78.7 ± 1.4	−0.5 ± 0.0	1.9 ± 1.0	4.0	PLA/VNC-D 1.5%	78.5 ± 0.9	−0.1 ± 0.1	−0.7 ± 0.4	2.4
PLA/BNC-D 3%	77.9 ± 1.1	−0.6 ± 0.0	1.9 ± 1.1	4.5	PLA/VNC-D 3%	76.4 ± 0.8	0.0 ± 0.0	0.8 ± 0.5	5.0
PLA/BNC-D 5%	79.9 ± 0.5	−0.5 ± 0.0	3.4 ± 0.4	5.0	PLA/VNC-D 5%	75.5 ± 1.2	0.2 ± 0.1	2.5 ± 0.8	6.7
PLA/BNC-D 7%	79.5 ± 0.5	−0.2 ± 0.2	6.2 ± 0.9	7.8	PLA/VNC-D 7%	73.9 ± 1.6	0.5 ± 0.2	4.4 ± 0.1	9.1
PLA/BNC-MB 0.5%	79.3 ± 1.1	−0.2 ± 0.0	−0.2 ± 0.5	2.0	PLA/VNC-MB 0.5%	80.7 ± 0.8	−0.2 ± 0.1	−1.8 ± 0.6	0.3
PLA/BNC-MB 1%	78.1 ± 0.5	−0.5 ± 0.0	1.4 ± 0.4	4.0	PLA/VNC-MB 1%	79.2 ± 0.8	−0.3 ± 0.0	0.4 ± 0.2	2.5
PLA/BNC-MB 1.5%	75.4 ± 1.5	−0.5 ± 0.0	3.4 ± 0.4	7.3	PLA/VNC-MB 1.5%	78.9 ± 1.2	−0.3 ± 0.0	0.4 ± 0.8	2.7
PLA/BNC-MB 3%	77.2 ± 0.9	−0.2 ± 0.2	7.3 ± 0.5	9.5	PLA/VNC-MB 3%	75.6 ± 1.3	0.1 ± 0.1	4.4 ± 0.8	7.9
PLA/BNC-MB 5%	78.1 ± 1.1	−0.2 ± 0.1	8.2 ± 0.9	10.1	PLA/VNC-MB 5%	69.3 ± 0.7	1.2 ± 0.0	10.2 ± 0.3	16.5
PLA/BNC-MB 7%	72.5 ± 1.8	2.0 ± 0.4	14.5 ± 0.8	18.2	PLA/VNC-MB 7%	68.8 ± 1.0	1.7 ± 0.3	11.6 ± 1.0	17.9
PLA/AcBNC-MB 0.5%	79.3 ± 0.5	−0.3 ± 0.4	−0.5 ± 0.5	1.8	PLA/AcVNC-MB 0.5%	81.7 ± 0.5	−0.3 ± 0.0	−1.4 ± 0.3	0.9
PLA/AcBNC-MB 1%	77.4 ± 1.2	0.2 ± 0.1	2.4 ± 1.0	5.2	PLA/AcVNC-MB 1%	81.1 ± 0.5	−0.4 ± 0.0	−1.4 ± 0.3	0.4
PLA/AcBNC-MB 1.5%	78.2 ± 0.9	−0.2 ± 0.0	0.6 ± 0.6	3.4	PLA/AcVNC-MB 1.5%	80.7 ± 0.7	−0.3 ± 0.0	−1.2 ± 0.3	0.3
PLA/AcBNC-MB 3%	73.6 ± 0.5	1.0 ± 0.1	7.7 ± 0.4	11.8	PLA/AcVNC-MB 3%	78.9 ± 0.5	−0.4 ± 0.0	0.5 ± 0.4	2.8
PLA/AcBNC-MB 5%	68.6 ± 1.6	1.8 ± 0.3	12 ± 0.8	18.3	PLA/AcVNC-MB 5%	77.2 ± 0.5	−0.3 ± 0.1	2.2 ± 0.4	5.2
PLA/AcBNC-MB 7%	66.0 ± 0.7	3.0 ± 0.1	15.3 ± 0.3	27.4	PLA/AcVNC-MB 7%	72.8 ± 0.3	0.0 ± 0.1	6.1 ± 0.3	11.0

## Data Availability

Data are contained within the article.
